# Serum Periostin as a Potential Biomarker in Pediatric Patients with Primary Hypertension

**DOI:** 10.3390/jcm10102138

**Published:** 2021-05-15

**Authors:** Michał Szyszka, Piotr Skrzypczyk, Anna Stelmaszczyk-Emmel, Małgorzata Pańczyk-Tomaszewska

**Affiliations:** 1 Department of Pediatrics and Nephrology, Doctoral School, Medical University of Warsaw, 02-091 Warsaw, Poland; michalszyszkaa@gmail.com; 2 Department of Pediatrics and Nephrology, Medical University of Warsaw, 02-091 Warsaw, Poland; mpanczyk1@wum.edu.pl; 3 Department of Laboratory Diagnostics and Clinical Immunology of Developmental Age, Medical University of Warsaw, 02-091 Warsaw, Poland; anna.stelmaszczyk-emmel@wum.edu.pl

**Keywords:** periostin, primary hypertension, children, adolescents, arterial damage, blood pressure

## Abstract

Experimental studies suggest that periostin is involved in tissue repair and remodeling. The study aimed to evaluate serum periostin concentration as potential biomarker in pediatric patients with primary hypertension (PH). We measured serum periostin, blood pressure, arterial damage, biochemical, and clinical data in 50 children with PH and 20 age-matched healthy controls. In univariate analysis, children with PH had significantly lower serum periostin compared to healthy peers (35.42 ± 10.43 vs. 42.16 ± 12.82 [ng/mL], *p* = 0.038). In the entire group of 70 children serum periostin concentration correlated negatively with peripheral, central, and ambulatory blood pressure, as well as with aortic pulse wave velocity (aPWV). In multivariate analysis, periostin level significantly correlated with age (β = −0.614, [95% confidence interval (CI), −0.831–−0.398]), uric acid (β = 0.328, [95%CI, 0.124–0.533]), body mass index (BMI) Z-score (β = −0.293, [95%CI, −0.492–−0.095]), high-density lipoprotein (HDL)-cholesterol (β = 0.235, [95%CI, 0.054–0.416]), and triglycerides (β = −0.198, [95%CI, −0.394–−0.002]). Neither the presence of hypertension nor blood pressure and aPWV influenced periostin level. To conclude, the role of serum periostin as a biomarker of elevated blood pressure and arterial damage in pediatric patients with primary hypertension is yet to be unmasked. Age, body mass index, uric acid, and lipid concentrations are key factors influencing periostin level in pediatric patients.

## 1. Introduction

Arterial hypertension (AH), one of the most common diseases worldwide, is a recognized risk factor for renal and cardiovascular diseases [1]. Its prevalence is constantly increasing, not only among adults, but also in the pediatric population. The increasingly common sedentary lifestyle, especially during the coronavirus disease 2019 (COVID-19) pandemic, as well as excessive salt intake accelerated the prevalence of AH. As recent studies show, AH is found in approximately 3–5% of all children [2]. Although in early childhood the majority of hypertension cases are secondary to other disorders, according to some new data, primary hypertension (PH) is taking the lead as the most common form of hypertension in children over 7 years of age [3]. PH emerges from the complex interactions of genetic and environmental factors. One of them is dysregulation of the renin–angiotensin–aldosterone system (RAAS), which plays a pivotal role in PH development [4].

Evaluation of arterial stiffness and common carotid artery intima-media thickness (cIMT) are included in the assessment of hypertension-mediated organ damage in children [5,6]. Increased cIMT and excessive arterial stiffness are common among pediatric patients with PH [7]. Furthermore, current observations and reviews point out that not only reduction of systolic and diastolic blood pressure (BP) should be taken into consideration as an aim in PH therapy, but also lowering pulse pressure and arterial stiffness are important independent treatment goals [8,9]. Oxidative stress, endothelial dysfunction, calcification, high collagen concentrations, and subclinical inflammation are linked to arterial wall dysfunction and its stiffening [10,11].

Periostin is a matricellular protein, a member of the fasciclin family [12]. It is produced mostly in utero and in differentiated connective tissues exposed to mechanical load such as aorta, heart valves, stomach, skin, tendons, and bones [13]. Periostin can also be upregulated after injury, and during remodeling and wound healing [14]. Experimental studies suggested a significant role of periostin in the development of arterial hypertension and hypertension-mediated organ damage. Firstly, periostin was found to interact with RAAS [15,16,17]; secondly, its expression in the arterial wall was elevated in a mice model of arterial hypertension [18]. Finally, periostin was found to be a promising marker of hypertension-induced cardiac remodeling [17] and hypertensive nephropathy [19]. Of note, treatment with RAAS-inhibiting agents led to significant down-regulation of tissue periostin level and reversal of kidney [19] and heart failure [20]. In addition, few human adult studies point to the role of periostin as a biomarker in cardiovascular and renal diseases. Periostin was revealed as a promising marker of diabetic kidney disease [21] and chronic allograft nephropathy [22] and increased periostin expression was found in patients with glomerulopathies [23]. Ling revealed that high serum periostin not only correlated with left ventricular ejection fraction but was also a marker of poor prognosis in patients with acute myocardial infarction (AMI) [24]. Nonetheless, little is known about the significance of the evaluation of periostin level in hypertensive patients.

We hypothesize that serum periostin could serve as a useful biomarker in hypertensive pediatric patients. Hence, the aims of our study were: 1. to compare serum periostin level in pediatric patients with primary hypertension and their healthy peers; 2. to reveal the relation between blood pressure (including central and 24-h ambulatory blood pressure), arterial damage (cIMT and increased arterial stiffness) and serum periostin 3. to reveal other significant determinants of serum periostin 4. to test serum periostin as a potential biomarker of subclinical arterial damage in children and adolescents with PH. 

## 2. Materials and Methods

### 2.1. Study Group 

**Figure 1:**
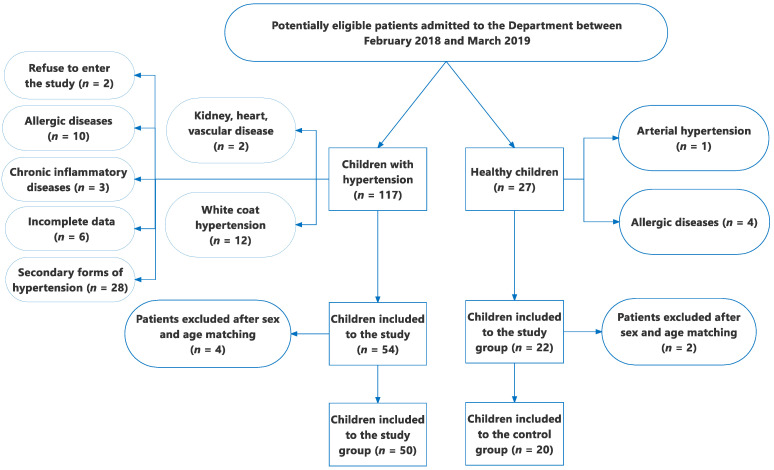
Flow diagram of the patients’ recruitment.

Sample size was estimated based on available literature on periostin with statistical power 0.8, *p* = 0.05, and effect size 0.55 should be ~50 [25,26,27,28,29,30,31,32,33,34,35]. Fifty pediatric patients with PH were recruited to this single-center cross-sectional study from patients hospitalized in a pediatric nephrology center between February 2018 and March 2019. Participants’ age varied from 5.58 to 17.92 years. The inclusion criterion was arterial hypertension diagnosed according to current European guidelines [5]. The exclusion criteria were secondary hypertension, clinically significant or laboratory confirmed allergic disease, known heart, renal, vascular, or other serious pathology, and acute inflammatory infections (temporary exclusion—4 weeks). 20 age-, and sex-matched healthy subjects were included in the control group. Their age varied from 8.33 to 17.83 years. The flow diagram of recruited patients is shown in Figure 1.

### 2.2. Ethical Issues

Approval of the Bioethical Committee of the Medical University of Warsaw was obtained before the initiation of the research (approval no. KB/58/2016, 15 March 2016). All procedures involving human participants were in accordance with the highest ethical standards of the institutional research committee and were performed in accordance with the Declaration of Helsinki on the treatment of human subjects and its later amendments. Informed consent was obtained from all participants (≥16 years) and their representatives included in the study.

### 2.3. Clinical Parameters

In all patients upon admission, we assessed basic anthropometric parameters including height (cm), weight (kg), and body mass index (BMI) (kg/m^2^). These measurements were compared with Polish normative data and expressed as Z-score [36]. In accordance with the World Health Organization definition, children with BMI Z-score values >1 and >2 were regarded as overweight and obese, respectively [37]. 

### 2.4. Serum Periostin

Venous peripheral blood was collected after overnight fasting. Next, blood was centrifuged to obtain serum, and stored at −80 °C until further analysis. Periostin levels were measured in serum samples by enzyme-linked immunosorbent assay (ELISA) (Human ELISA kit, Cat. No. RAG019R (ng/mL), Biovendor, Brno, Czech Republic) and the results were read using Biochrom Asys UVM 340 Scanning Microplate Reader (Biochrom Ltd., Cambridge, UK).

### 2.5. Other Laboratory Tests

Other parameters measured from peripheral blood in all children included: number of neutrophils (NEU; 1000/μL), lymphocytes (LYM; 1000/μL), platelets (PLT; 1000/μL), mean platelet volume (MPV; fL), and neutrophil-to-lymphocyte as well as platelet-to-lymphocyte ratios (NLR and PLR, respectively), serum creatinine (mg/dL), uric acid (mg/dL), total, low density lipoprotein (LDL), high-density lipoprotein (HDL) cholesterol (mg/dL), and triglycerides (mg/dL), calcium (mg/dL), phosphate (mg/dL), parathormone (PTH; pg/mL), alkaline phosphatase (IU/L), and vitamin D concentration (25(OH)D) (ng/mL). Glomerular filtration rate was estimated according to the revised Schwartz formula (eGFR) (mL/min/1.73 m^2^) [38].

### 2.6. Blood Pressure and Arterial Damage Parameters

In all patients with PH, the duration of hypertension was estimated based on medical records and in those receiving pharmacological treatment antihypertensive medications were analyzed. The BP measurement methodology and arterial damage assessment were described in detail in our previous manuscripts [39,40,41,42]. In short, peripheral office BP was evaluated oscillometrically by Welch Allyn VSM Patient Monitor 300 (Welch Allyn Inc., Skaneateles Falls, NY, USA) ((mmHg) and Z-scores) [43]. We performed 24 h ambulatory blood pressure monitoring (ABPM) with SUNTECH OSCAR 2 oscillometric device (SunTech Medical, Inc., Morrisville, NC, USA) and interpreted according to pediatric recommendations [44] with the following parameters included in the final analysis: systolic, diastolic, and mean arterial pressure (SBP, DBP, MAP, respectively) during 24 h (mm Hg), MAP 24 h Z-score, pulse pressure (mm Hg), heart rate (beats per minute), SBP and DBP load during 24 h (%), and nocturnal blood pressure dip (%) [44]. Central (aortic) blood pressure (AoBP) (mm Hg), augmentation index normalized to heart rate of 75 beats per minute (Aix75HR) (%), subendocardial viability ratio (SEVR) (%), and aortic pulse wave velocity (aPWV) (m/s) were measured using the Sphygmocor device (AtCor Medical Pty Ltd., Sydney, Australia) and applanation tonometry. Aloka Prosound Alpha 6 (Hitachi Aloka Medical, Mitaka, Japan) equipped with a 13 MHz linear transducer was used to measure common carotid intima media thickness (cIMT) (mm) and elasticity parameters of the right common carotid artery (ECHO-tracking (ET) preset): beta (stiffness index), Ep (pressure strain elasticity modulus) (kPa), AC (arterial compliance) (mm^2^/kPa), AIx (augmentation index) (%), PWVbeta (pulse wave velocity beta) (m/s), D_max (mm), D_min (mm) (maximal and minimal diameter of the artery), DATmax (acceleration time to artery maximal diameter) (ms). aPWV and cIMT were presented as numeric values and Z-scores [45,46].

### 2.7. Statistical Analysis

Statistical data were analyzed using Dell Statistica 13.0 PL software (TIBCO Software Inc., Palo Alto, CA, USA). All data were reported as absolute numbers, mean ± standard deviation (SD), and interquartile range (IQR). Normality of data distribution was analyzed with the Shapiro–Wilk test. The following tests were used (depending upon variables’ distribution): Student *t*-test, U Mann–Whitney test, Spearman rank correlation, chi-square test, and Fisher’s exact test. Multivariate analysis was performed using a general regression model. Parameters that correlated with each other with r >0.600 were excluded from the regression model to avoid collinearity. A *p*-value below 0.05 was considered as statistically significant.

## 3. Results

### 3.1. Periostin, Clinical and Laboratory Parameters

Both groups’ basic clinical parameters and results of key laboratory tests, including periostin levels, were depicted in Table 1. PH and healthy children did not differ in terms of age, sex, height, height Z-score, eGFR, and total-, and LDL- cholesterol. Periostin levels were significantly lower in hypertensive children compared to healthy ones (*p* = 0.038). Children with PH were characterized also by significantly higher weight, BMI, concentrations of uric acid, and triglycerides. HDL-cholesterol levels were significantly lower in the study group compared to healthy children. In the PH group 21 (42%) of all patients were overweight and 7 (14%) were obese. The duration of hypertension in the study group was 18.14 ± 20.86 (4–24) (months). At the moment of evaluation, 27 (54.0%) children received pharmacological antihypertensive treatment—23 were on monotherapy, 3 were treated with 2 medications, and one child received 3 medications. Most commonly used agents were as follows: calcium channel blockers (17 children), angiotensin-converting enzyme inhibitors (9 children), and beta-adrenolytics (3 children). In addition, 3 children were treated with doxazosin, valsartan, and hydrochlorothiazide. Parameters of complete blood count and calcium-phosphate metabolism were presented in Supplementary Table S1.

### 3.2. Blood Pressure and Parameters of Arterial Damage

The comparison of peripheral and central blood pressure, arterial stiffness, and intima media thickness in the study and control group are shown in Supplementary Tables S2 and S3. 

Children and adolescents with PH had higher office peripheral and central blood pressures, as well as blood pressure measured with ABPM. There was no difference in 24 h ABPM heart rate and pulse pressure (PP), and aortic pulse pressure (AoPP) and both systolic and diastolic nocturnal BP dipping between both groups. Patients with PH were characterized by faster aortic pulse wave velocity (5.17 ± 0.93 vs. 4.49 ± 0.72 (m/s), *p* = 0.004), thicker common carotid artery intima media layer (0.45 ± 0.07 vs. 0.39 ± 0.03 (mm), *p* < 0.001), and larger common carotid artery (maximal and minimal) diameters. No differences were found in aortic augmentation index, subendocardial viability ratio, and local stiffness parameters.

### 3.3. Determinants of Serum Periostin Level

Periostin level did not differ between PH patients either on or off antihypertensive drugs (34.89 ± 9.86 (28.80–37.89) vs. 36.00 ± 11.36 (27.22–41.10) (ng/mL), *p* = 0.954) and between 10 PH patients treated and 40 patients not treated with renin-angiotensin-aldosterone system inhibitors (34.96 ± 6.47 (30.77–37.41) vs. 35.54 ± 11.27 (27.42–40.20) (ng/mL), *p* = 0.602). Additionally, no difference between hypertensive boys and girls was found (34.89 ± 9.86 (28.80–37.89) vs. 36.00 ± 11.36 (27.22–41.10) (ng/mL), *p* = 0.954).

The correlations of periostin with clinical and laboratory parameters in the whole group of 70 children are shown in Table 2. We found positive correlations of periostin concentration with HDL-cholesterol and phosphate, calcium-phosphate product, and alkaline phosphatase activity. Negative correlations of periostin with age, height, weight, BMI, NLR, and concentrations of creatinine, uric acid, and triglycerides were observed. Furthermore, periostin level correlated negatively with numerous indices of blood pressure, aortic pulse wave velocity, and common carotid artery diameters. The correlations of periostin with the studied parameters in separate groups are shown in Table 3 and Table 4 for the study and the control group, respectively. In 50 children with PH periostin correlated positively with height Z-score, number of lymphocytes, calcium, phosphate, calcium-phosphate product, and alkaline phosphatase, and negatively with age, weight, and BMI. These three anthropometrical parameters as well as serum creatinine, PWV, PWV Z-score, office SBP, AoSBP (aortic systolic blood pressure) were also negatively correlated with periostin in 20 healthy children. In the control group periostin also correlated positively with HDL-cholesterol, phosphate, alkaline phosphatase, and AIx75HR. No significant correlation between serum periostin and eGFR in the whole group, and in patients with primary hypertension, and control group were found.

To verify independent associations between periostin, blood pressure, and arterial stiffness, multivariate analysis was performed (Table 5). As shown in Table 5, age, BMI Z-score, uric acid, HDL-cholesterol, and triglycerides were the significant independent determinants of periostin concentration in children.

## 4. Discussion

Our cross-sectional study analyzed periostin as a possible biomarker of blood pressure and subclinical arterial damage in pediatric patients with primary hypertension. Univariate analysis showed that periostin level was lower in hypertensive individuals as compared to the control group. Moreover, it revealed numerous negative correlations between periostin level, blood pressure, and arterial damage parameters. Nevertheless, these relations disappeared in multivariate analysis leaving only the following significant predictors of periostin: age, BMI Z-score, uric acid, HDL-cholesterol, and triglycerides. In multivariate analysis, neither the presence of hypertension nor blood pressure significantly influenced periostin level. 

Periostin was found to be involved in tissue repair after vascular injury, e.g., in acute rheumatic fever [28], hypertensive nephropathy [19], myocardial infarction [47], and subarachnoid hemorrhage [48]. Periostin plays a critical role in the interaction with signaling proteins such as NF-kB (nuclear factor kappa B) or STAT3 (signal transducer and activator of transcription) to modulate the response of the extracellular matrix in various tissue pathologies [28]. It is hypothesized that after injury periostin expression increases and thus facilitates tissue repair and remodeling. A cross-talk between periostin and transforming growth factor beta (TGFβ) signals in different tissues and pathological conditions was described [49]. This mutual, reciprocal relation was revealed e.g., in scleroderma and allergic diseases [50]. Periostin augments adhesion and TGFβ release in immune cells. Reciprocally, TGFβ induces periostin production in fibroblasts [50]. Similar interplay was revealed in kidney tissue, where periostin can induce cell dedifferentiation, increase in TGFβ expression and extracellular matrix deposition. In addition, TGFβ can also promote the expression of periostin, which, in turn, induces the loss of renal tubular epithelial phenotype (epithelial-mesenchymal transition) and ultimately leads to fibrosis [51].

It is noteworthy that elevated expression of periostin in the arterial wall was found in a hypoxia-induced model of pulmonary hypertension [52]. Moreover, periostin played a pivotal role in aortal thickening in hypertensive rats [18]. Hence, one would expect a higher periostin level in hypertensive patients and its positive correlation with arterial damage parameters. Unintuitively, in our hypertensive patients periostin level was lower and correlated negatively with both blood pressure and aortic pulse wave velocity. Notably, these relations disappeared in multivariate analysis. It is possible that periostin is not released yet in the subclinical damage found in our patients or serum periostin concentrations do not correspond with tissue (arterial wall) periostin expression. Further studies are needed to elucidate the role of periostin in the development of early stages of hypertensive diseases and vascular damage in these patients. 

Interleukins (ILs) 3, 4, 6, 13, tumor necrosis factor alpha (TNFα), TGFβ, and vascular endothelial growth factor (VEGF) are the best known inducers of periostin expression and release [53]. In particular, increased IL-4 and IL-13 were found to be responsible for high periostin levels in allergic children [26] and TNFα and IL-6 in obese adults [33]. That is why the patients with chronic inflammatory diseases, allergies, and acute infections were excluded from the analysis to avoid the impact of these comorbidities on the periostin level. However, nowadays arterial hypertension is considered a state of subclinical inflammation with numerous inflammatory markers elevated that may link PH with dysregulation of periostin level [54]. Our PH children had higher neutrophil count, lower platelet volume, and a trend towards a higher neutrophil-to-lymphocyte ratio as compared to healthy peers. Univariate analysis showed a negative correlation between NLR and periostin level, but this relation was excluded in the multivariate analysis. Based on these results, no definite statement on the mutual relation between subclinical inflammation and periostin level in PH patients can be made. Analysis of the relation between periostin and more precise markers of inflammation (e.g., high-sensitivity C-reactive protein or interleukin levels) in patients with PH would be of special interest.

Activation of the renin–angiotensin–aldosterone system could be a link between periostin and cardiovascular system regulation. One study showed increased periostin levels in rats in response to chronic infusion of angiotensin II [16]. Other paper points out that periostin can contribute to oxidative stress and is upregulated by angiotensin II via the reactive oxygen species signaling pathway in fibroblasts of hypertensive rats [17]. The mutual relation between RAAS and periostin may be more complex as periostin downregulation attenuated 5/6 nephrectomy-induced intrarenal RAAS activation and renal tissue fibrosis [15]. We did not measure plasma renin activity or aldosterone levels in the studied subjects. Twenty percent of our hypertensive patients were on RAAS blockade and no difference was found between those treated and not treated with angiotensin receptor blockers and angiotensin converting enzyme inhibitors. 

Age was the strongest predictor of periostin level in our cohort. Limited studies showed decrease in periostin levels with age [34,35]. Similarly to our observations, O’Connell at al. found a negative relation between periostin level and age. Of note, these authors analyzed only children aged less than two years [34]. Elevated periostin levels seen in younger children may reflect the accelerated cell turnover and growth in the first few years of life and which would naturally increase periostin expression. Negative correlations of periostin, blood pressure, and pulse wave velocity were absent in multivariate analysis, as these parameters are strongly related to age and BMI in the pediatric population [36,43,45].

Epidemiological studies show an association between cardiovascular diseases, hypertension, metabolic syndrome, and high levels of uric acid. In addition, some data suggest that high uric acid levels can predict the development of hypertension [55]. Animal studies revealed positive correlations between uric acid concentration, inflammation, and decreased expression of neuronal nitric oxide synthase, which results in blood vessel contraction [56]. Additionally, in our cohort almost half of hypertensive subjects presented with hyperuricemia. Our multivariate analysis revealed a positive correlation between uric acid and periostin levels. A similar positive relation was found in Chinese adult women with polycystic ovary syndrome [29]. It is possible that periostin is released in response to subclinical damage caused by uric acid elevation.

In univariate analysis, serum periostin inversely correlated with serum creatinine, which could seemingly indicate the dependence of this marker on renal function. However, it should be noted that patients in both groups had normal renal function (eGFR > 60 mL/min/1.73 m^2^ according to Schwartz’s formula, which is known to underestimate glomerular filtration rate in adolescents [38,57]). Serum creatinine concentration is dependent on weight and age, hence this apparent relationship disappeared in the multivariate analysis. We did not observe any relationship between periostin and eGFR in the children studied. Published data on mutual relation between kidney function and serum periostin level are scarce. A trend towards positive correlation between creatinine and serum periostin was found in adults with diabetic kidney disease [58]. On the other hand, urinary periostin was negatively correlated with eGFR in adult patients with diabetic kidney disease [21] and chronic allograft nephropathy [22]. Also, increased glomerular periostin staining was related to low eGFR in different glomerulopathies [23].

The results of our multivariate analysis demonstrated that periostin level is positively correlated with HDL-cholesterol and negatively with triglyceride concentration as well as with BMI Z-score. HDLs are characterized by a well-established protective effect on the arterial wall and their negative correlations with cardiovascular incidents are well documented [59]. There is conflicting data concerning the role of periostin in lipid metabolism. By contrast with our results, in two Chinese studies involving young women with polycystic ovary syndrome [29] and adult obese patients with type 2 diabetes [33], periostin concentration was directly correlated with BMI and triglycerides and inversely correlated with HDL-cholesterol. Lu et al. found that overexpression of periostin in obese rats resulted in liver steatosis and hypertriglyceridemia via activation of c-Jun N-terminal kinase (JNK) signaling pathway and downregulation of peroxisome proliferator-activated receptor alpha (PPARα) [60]. On the other hand, a recent interventional study in rats after myocardial infarction showed a beneficial effect of periostin supply on HDL-cholesterol [47]. These experimental results suggest the positive impact of the studied particle on the regulation of cholesterol and triglycerides levels. 

The cross-sectional nature of the study that precludes drawing final casual relationships between the measured parameters is a major limitation of our study. A low number of patients in the control group is another disadvantage. The large heterogeneity in age and BMI of the patients resulted in the demonstration of numerous correlations in univariate analysis that proved to be statistically insignificant in multivariate analysis (e.g., creatinine). Of note, our analysis of immune system activation was limited only to low-precision parameters derived from peripheral complete blood count. In addition, we have not evaluated concentrations of other key players in pathogenesis of tissue damage and repair e.g., TGFβ. Finally, neither urine nor tissue (vascular wall) periostin levels were analyzed in the studied children. Of note, deep analysis of blood pressure and general and local (carotid artery) vascular damage are particular strengths of the study.

## 5. Conclusions

The role of serum periostin as a biomarker of elevated blood pressure and arterial damage in pediatric patients with primary hypertension is yet to be unmasked. More clinical studies are needed to reveal the changes of periostin in hypertensive patients’ serum and urine, and to clarify its role in this population. Age seems to be the strongest predictor of serum periostin level in pediatric population. We have not found any significant dependences of serum periostin with blood pressure and arterial damage analyzed according to different aspects. On the other hand, there was a significant relation of serum periostin with well-established cardiovascular risk factors. Considering young age of our patients and relatively short duration of PH as compared to adults, it is possible that these correlations have not markedly influenced blood pressure and target organs in children with PH yet.

## Figures and Tables

**Table 1:** Clinical and biochemical parameters of the study and the control group (data presented as numbers or mean ± standard deviation and interquartile range).

Parameter	Study Group	Control Group	*p*
Number of patients (*n*)	50	20	NA
Age (years)	14.76 ± 3.08 (14.42–16.75)	14.11 ± 2.99 (13.00–16.38)	0.261
Boys/Girls	29/21 (58%/42%)	11/9 (55%/45%)	0.819
Height (cm)	166.7 ± 16.51 (160.5–178.5)	161.9 ± 15.26 (151.5–171.5)	0.259
Height Z-score	0.54 ± 0.99 (−0.45–1.16)	0.69 ± 1.23 (0.13–1.22)	0.589
Weight (kg)	70.29 ± 19.6 (61.5–85.0)	55.2 ± 16.55 (46.0–61.0)	0.003
Weight Z-score	1.23 ± 0.80 (0.49–1.83)	0.61 ± 0.98 (−0.26–1.25)	0.009
BMI	24.78 ± 4.63 (21.27–28.89)	20.67 ± 3.59 (18.30–22.03)	0.001
BMI Z-score	1.09 ± 0.82 (0.5–1.78)	0.35 ± 0.94 (−0.43–1.12)	0.004
Periostin (ng/mL)	35.42 ± 10.43 (27.73–39.02)	42.16 ± 12.82 (32.21–51.45)	0.038
eGFR acc. to Schwartz (L/min/1.73 m^2^)	100.12 ± 21.16 (86.73–115.64)	108.12 ± 18.48 (95.42–121.9)	0.100
Uric acid (mg/dL)	5.56 ± 1.34 (4.7–6.4)	4.56 ± 1.10 (3.95–5.35)	0.004
Total cholesterol (mg/dL)	153.98 ± 33.92 (132.0–168.0)	154.75 ± 30.71 (135.0–179.5)	0.927
LDL-cholesterol (mg/dL)	85.63 ± 30.38 (64.0–95.8)	81.2 ± 24.19 (63.8–103.6)	0.654
HDL-cholesterol (mg/dL)	49.5 ± 10.28 (43.0–53.0)	61.05 ± 13.05 (52.5–69.5)	0.001
Triglycerides (mg/dL)	95.24 ± 43.18 (64.0–120.0)	64.5 ± 23.86 (49.5–71.5)	0.002

NA: not applicable; BMI: body mass index; eGFR: estimated glomerular filtration rate; LDL: low-density lipoprotein; HDL: high-density lipoprotein.

**Table 2:** Correlations of periostin concentration with clinical, biochemical parameters, blood pressure, and arterial damage parameters in 70 studied children.

Analyzed Parameter	R	*p*
Periostin (ng/mL) vs. age (years)	−0.561	<0.001
Periostin (ng/mL) vs. height Z-score	−0.278	0.020
Periostin (ng/mL) vs. weight (kg)	−0.505	<0.001
Periostin (ng/mL) vs. BMI (kg/m^2^)	−0.585	<0.001
Periostin (ng/mL) vs. BMI Z-score	−0.298	0.012
Periostin (ng/mL) vs. NLR	−0.270	0.024
Periostin (ng/mL) vs. creatinine (mg/dL)	−0.301	0.011
Periostin (ng/mL) vs. uric acid (mg/dL)	−0.240	0.045
Periostin (ng/mL) vs. HDL-cholesterol (mg/dL)	0.397	<0.001
Periostin (ng/mL) vs. triglycerides (mg/dL)	−0.245	0.041
Periostin (ng/mL) vs. phosphate (mg/dL)	0.421	<0.001
Periostin (ng/mL) vs. Ca * P (mg^2^/dL^2^)	0.423	<0.001
Periostin (ng/mL) vs. alkaline phosphatase (IU/L)	0.694	<0.001
Periostin (ng/mL) vs. SBP (mm Hg)	−0.370	0.004
Periostin (ng/mL) vs. SBP Z-score	−0.269	0.024
Periostin (ng/mL) vs. DBP (mm Hg)	−0.303	0.011
Periostin (ng/mL) vs. DBP Z-score	−0.323	0.006
Periostin (ng/mL) vs. MAP (mm Hg)	−0.320	0.007
Periostin (ng/mL) vs. ABPM SBP 24 h (mm Hg)	−0.285	0.017
Periostin (ng/mL) vs. ABPM MAP 24 h (mm Hg)	−0.251	0.036
Periostin (ng/mL) vs. ABPM PP 24 h (mm Hg)	−0.243	0.043
Periostin (ng/mL) vs. AoSBP (mm Hg)	−0.340	0.004
Periostin (ng/mL) vs. AoDBP (mm Hg)	−0.333	0.005
Periostin (ng/mL) vs. AoMAP (mm Hg)	−0.334	0.005
Periostin (ng/mL) vs. aPWV (m/s)	−0.342	0.004
Periostin (ng/mL) vs. aPWV Z-score	−0.306	0.010
Periostin (ng/mL) vs. ET D max (mm)	−0.379	0.001
Periostin (ng/mL) vs. ET D min (mm)	−0.353	0.003

BMI: body mass index; NLR: neutrophil-to-lymphocyte ratio; HDL: high-density lipoprotein; Ca * P: calcium phosphate product; SBP: systolic blood pressure; DBP: diastolic blood pressure; MAP: mean arterial pressure; PP: pulse pressure; ABPM: ambulatory blood pressure; HR: heart rate; bpm: beats per minute; SBPL: systolic blood pressure load; DBPL: diastolic blood pressure load; AoSBP: aortic (central) systolic blood pressure; AoDBP: aortic (central) diastolic blood pressure; AoMAP: aortic (central) mean blood pressure; aPWV: aortic pulse wave velocity; ET: ECHO-tracking; beta: stiffness index; D max: maximal diameter of the right common carotid artery; D min: minimal diameter of the right common carotid artery.

**Table 3:** Correlations of periostin concentration with clinical, biochemical parameters, blood pressure, and arterial damage parameters in 50 children with primary hypertension.

Analyzed Parameter	R	*p*
Periostin (ng/mL) vs. Age (years)	−0.554	<0.001
Periostin (ng/mL) vs. Height Z-score	0.325	0.021
Periostin (ng/mL) vs. Weight (kg)	−0.388	0.005
Periostin (ng/mL) vs. BMI (kg/m^2^)	−0.471	0.001
Periostin (ng/mL) vs. BMI Z-score	−0.194	0.177
Periostin (ng/mL) vs. NLR	−0.181	0.209
Periostin (ng/mL) vs. Creatinine (mg/dL)	−0.138	0.341
Periostin (ng/mL) vs. Uric acid (mg/dL)	−0.103	0.477
Periostin (ng/mL) vs. HDL-cholesterol (mg/dL)	0.239	0.094
Periostin (ng/mL) vs. Triglycerides (mg/dL)	−0.092	0.524
Periostin (ng/mL) vs. Phosphate (mg/dL)	0.369	0.008
Periostin (ng/mL) vs. Ca * P (mg^2^/dL^2^)	0.447	0.001
Periostin (ng/mL) vs. Alkaline Phosphatase (IU/L)	0.651	<0.001
Periostin (ng/mL) vs. SBP (mm Hg)	−0.208	0.148
Periostin (ng/mL) vs. SBP Z-score	−0.031	0.832
Periostin (ng/mL) vs. DBP (mm Hg)	−0.207	0.149
Periostin (ng/mL) vs. DBP Z-score	−0.205	0.153
Periostin (ng/mL) vs. MAP (mm Hg)	−0.208	0.147
Periostin (ng/mL) vs. ABPM SBP 24 h (mm Hg)	−0.182	0.205
Periostin (ng/mL) vs. ABPM MAP 24 h (mm Hg)	−0.134	0.352
Periostin (ng/mL) vs. ABPM PP 24 h (mm Hg)	−0.138	0.340
Periostin (ng/mL) vs. AoSBP (mm Hg)	−0.182	0.205
Periostin (ng/mL) vs. AoDBP (mm Hg)	−0.255	0.074
Periostin (ng/mL) vs. AoMAP (mm Hg)	−0.220	0.125
Periostin (ng/mL) vs. aPWV (m/s)	−0.226	0.115
Periostin (ng/mL) vs. aPWV Z-score	−0.172	0.233
Periostin (ng/mL) vs. ET D max (mm)	−0.276	0.053
Periostin (ng/mL) vs. ET D min (mm)	−0.244	0.088

BMI: body mass index; NLR: neutrophil-to-lymphocyte ratio; HDL: high-density lipoprotein; Ca * P: calcium phosphate product; SBP: systolic blood pressure; DBP: diastolic blood pressure; MAP: mean arterial pressure; PP: pulse pressure; ABPM: ambulatory blood pressure; HR: heart rate; bpm: beats per minute; SBPL: systolic blood pressure load; DBPL: diastolic blood pressure load; AoSBP: aortic (central) systolic blood pressure; AoDBP: aortic (central) diastolic blood pressure; AoMAP: aortic (central) mean blood pressure; aPWV: pulse wave velocity; ET: ECHO-tracking; beta: stiffness index; D max: maximal diameter of right common carotid artery; D min: minimal diameter of right common carotid artery.

**Table 4:** Correlations of periostin concentration with clinical, biochemical parameters, blood pressure, and arterial damage parameters in 20 healthy children.

Analyzed Parameter	R	*p*
Periostin (ng/mL) vs. Age (years)	−0.570	0.009
Periostin (ng/mL) vs. Height Z-score	0.198	0.403
Periostin (ng/mL) vs. Weight (kg)	−0.633	0.003
Periostin (ng/mL) vs. BMI (kg/m^2^)	−0.734	<0.001
Periostin (ng/mL) vs. BMI Z-score	−0.324	0.163
Periostin (ng/mL) vs. NLR	−0.277	0.238
Periostin (ng/mL) vs. Creatinine (mg/dL)	−0.474	0.035
Periostin (ng/mL) vs. Uric acid (mg/dL)	−0.108	0.651
Periostin (ng/mL) vs. HDL-cholesterol (mg/dL)	0.514	0.021
Periostin (ng/mL) vs. Triglycerides (mg/dL)	−0.351	0.130
Periostin (ng/mL) vs. Phosphate (mg/dL)	0.552	0.012
Periostin (ng/mL) vs. Ca * P (mg^2^/dL^2^)	0.416	0.068
Periostin (ng/mL) vs. Alkaline Phosphatase (IU/L)	0.693	0.001
Periostin (ng/mL) vs. SBP (mm Hg)	−0.454	0.044
Periostin (ng/mL) vs. SBP Z-score	−0.444	0.050
Periostin (ng/mL) vs. DBP (mm Hg)	−0.369	0.110
Periostin (ng/mL) vs. DBP Z-score	−0.277	0.238
Periostin (ng/mL) vs. MAP (mm Hg)	−0.361	0.118
Periostin (ng/mL) vs. ABPM SBP 24 h (mm Hg)	−0.078	0.742
Periostin (ng/mL) vs. ABPM MAP 24 h (mm Hg)	−0.156	0.512
Periostin (ng/mL) vs. ABPM PP 24 h (mm Hg)	0.018	0.939
Periostin (ng/mL) vs. AoSBP (mm Hg)	−0.477	0.034
Periostin (ng/mL) vs. AoDBP (mm Hg)	−0.328	0.158
Periostin (ng/mL) vs. AoMAP (mm Hg)	−0.361	0.118
Periostin (ng/mL) vs. PWV (m/s)	−0.462	0.040
Periostin (ng/mL) vs. PWV Z-score	−0.448	0.048
Periostin (ng/mL) vs. ET D max (mm)	−0.350	0.130
Periostin (ng/mL) vs. ET D min (mm)	−0.341	0.141

BMI: body mass index; NLR: neutrophil-to-lymphocyte ratio; HDL: high-density lipoprotein; Ca * P: calcium phosphate product; SBP: systolic blood pressure; DBP: diastolic blood pressure; MAP: mean arterial pressure; PP: pulse pressure; ABPM: ambulatory blood pressure; HR: heart rate; bpm: beats per minute; SBPL: systolic blood pressure load; DBPL: diastolic blood pressure load; AoSBP: aortic (central) systolic blood pressure; AoDBP: aortic (central) diastolic blood pressure; AoMAP: aortic (central) mean blood pressure; PWV: pulse wave velocity; ET: ECHO-tracking; beta: stiffness index; D max: maximal diameter of right common carotid artery; D min: minimal diameter of right common carotid artery.

**Table 5:** Multivariate analysis of periostin determinants in children.

Parameter	Βeta	95% Confidence Interval	*p*
Age (years)	−0.614	−0.831–(−0.398)	<0.001
Uric acid (mg/dL)	0.328	0.124–0.533	0.002
BMI Z-score	−0.293	−0.492–(−0.095)	0.005
HDL-cholesterol (mg/dL)	0.235	0.054–0.416	0.012
Triglycerides (mg/dL)	−0.198	−0.394–(−0.002)	0.048
DBP Z-score	−0.205	−0.434–0.025	0.079
Presence of hypertension (yes/no)	0.219	−0.032–0.469	0.087
ET D max (mm)	−0.149	−0.330–0.031	0.104
AoSBP (mm Hg)	−0.142	−0.381–0.097	0.240
Ca * P (mg^2^/dL^2^)	0.066	−0.119–0.251	0.478
NLR	−0.062	−0.245–0.121	0.499
Height Z-score	0.052	−0.124–0.229	0.555
aPWV Z-score	0.024	−0.162–0.210	0.795

BMI: body mass index; HDL: high-density lipoprotein; DBP: diastolic blood pressure; ET: ECHO-tracking; beta: stiffness index; D max: maximal diameter of the right common carotid artery; AoSBP: aortic (central) systolic blood pressure; Ca * P: calcium phosphate product; NLR: neutrophil-to-lymphocyte ratio; aPWV: aortic pulse wave velocity.

## Data Availability

Data used to support the findings of this study are included within the supplementary information files, Supplementary Table S4 (patient_data.xlsx).

## Supplementary Materials

The following are available online at https://www.mdpi.com/article/10.3390/jcm10102138/s1, Table S1: Parameters of complete blood count and parameters of calcium-phosphate metabolism of the study and the control group (data presented as mean ± standard deviation and interquartile range)., Table S2: Blood pressure in the study and the control group (data presented as numbers or mean ± standard deviation and interquartile range), Table S3: Parameters of arterial structure and function in the study and the control group (data presented as numbers or mean ± standard deviation and interquartile range). Table S4: Patient data (patient_data.xlsx).
