# Supplementary material for: Serum Periostin as a Potential Biomarker in Pediatric Patients with Primary Hypertension
Source: J Clin Med. 2021 May 15;10(10):2138. doi: 10.3390/jcm10102138 (PMC8156565; doi:10.3390/jcm10102138)
Supplement: Supplementary file 1 [file jcm-10-02138-s001.zip › jcm-1192343-supplementary/Supplementary Table S1.pdf]

Journal of
Clinical Medicine

MDPI

Supplementary Table S1. Parameters of complete blood count and parameters of calcium-phosphate metabolism of the study and the control group (data presented as mean ± standard deviation and interquartile range).

Parameter	Study group	Control group	*p*
Neutrophils [1000/µL]	3.83 ± 1.85 (2.61 – 4.58)	2.73 ± 0.83 (1.86 – 3.07)	0.004
Lymphocytes [1000/µL]	2.3 ± 0.74 (1.9 – 2.68)	2.16 ± 0.41 (1.82 – 2.55)	0.516
Platelets [1000/µL]	237.8 ± 77.74 (223.0 – 324.0)	248.5 ± 46.47 (217 – 287.5)	0.179
MPV [fL]	10.19 ± 1.18 (9.7 – 11.1)	10.91 ± 0.83 (10.2 – 11.4)	0.032
NLR	1.91 ± 1.44 (1.17 – 1.99)	1.27 ± 0.48 (0.89 - 1.53)	0.066
PLR	126.61 ± 40.34 (98.18 – 148.56)	118.94 ± 29.28 (96.77 – 135.11)	0.443
Calcium [mg/dL]	9.99 ± 0.35 (9.8 – 10.3)	9.77 ± 0.30 (9.6 – 10.0)	0.014
Phosphate [mg/dL]	4.40 ± 0.70 (3.9 – 4.8)	4.50 ± 0.43 (4.2 – 4.8)	0.585
Ca * P [mg^2/dL^2]	43.98 ± 7.42 (38.61 – 48.41)	43.85 ± 3.87 (41.26 – 46.54)	0.941
Parathormone [pg/mL]	29.43 ± 16.99 (16.8 – 36.4)	23.19 ± 10.14 (14.9 – 31.2)	0.198
Alkaline phosphatase [IU/L]	122.26 ± 54.75 (76 – 153)	151.90 ± 71.19 (93 – 197)	0.095
25(OH)D [ng/mL]	19.47 ± 8.06 (15.50 – 22.20)	21.71 ± 9.10 (15.75 – 25.85)	0.398

MPV: mean platelet volume; NLR: neutrophil-to-lymphocyte ratio; PLR: platelet-to-lymphocyte ratio; Ca * P: calcium phosphate product; 25(OH)D: 25-hydoxy-vitamin D.
