# Supplementary material for: Serum Periostin as a Potential Biomarker in Pediatric Patients with Primary Hypertension
Source: J Clin Med. 2021 May 15;10(10):2138. doi: 10.3390/jcm10102138 (PMC8156565; doi:10.3390/jcm10102138)
Supplement: Supplementary file 1 [file jcm-10-02138-s001.zip › jcm-1192343-supplementary/Supplementary Table S2.pdf]

Journal of
Clinical Medicine

MDPI

Supplementary Table S2. Blood pressure in the study and the control group (data presented as mean ± standard deviation and interquartile range).

Parameter	Study group	Control group	*p*
Office SBP<br>[mm Hg]	131.50 ± 12.36<br>(122.00 – 140.00)	117.00 ± 9.90<br>(109.00 – 125.00)	<0.001
Office SBP<br>Z-score	2.30 ± 0.92<br>(1.68 – 2.79)	0.65 ± 0.86<br>(0.01 – 1.41)	<0.001
Office DBP<br>[mm Hg]	78.82 ± 10.50<br>(73.00 – 83.00)	66.40 ± 6.48<br>(62.00 – 70.00)	<0.001
Office DBP<br>Z-score	1.59 ± 0.85<br>(1.09 – 2.03)	0.18 ± 0.67<br>(−0.31 – 0.75)	<0.001
Office MAP<br>[mm Hg]	95.28 ± 10.25<br>(87.67 – 101.33)	82.30 ± 6.58<br>(76.33 – 87.17)	<0.001
Office PP<br>[mm Hg]	52.69 ± 10.44<br>(45.00 – 61.00)	50.60 ± 7.21<br>(45.0 – 55.0)	0.415
ABPM SBP 24h<br>[mm Hg]	127.88 ± 8.37<br>(121.00 – 133.00)	112.95 ± 6.06<br>(110.00 – 116.50)	<0.001
ABPM DBP 24h<br>[mm Hg]	70.44 ± 6.41<br>(66.00 – 73.00)	63.20 ± 3.85<br>(60.50 – 67.00)	<0.001
ABPM MAP 24h<br>[mm Hg]	89.48 ± 6.40<br>(85.00 – 93.00)	79.65 ± 3.54<br>(77.00 – 82.50)	<0.001
ABPM MAP 24<br>Z-score	1.16 ± 1.40<br>(0.17 – 1.61)	−0.44 ± 0.66<br>(−0.87 – 0.08)	<0.001
ABPM PP 24h<br>[mm Hg]	57.54 ± 7.04<br>(52.00 – 62.00)	50.00 ± 6.21<br>(46.50 – 52.50)	<0.001
ABPM HR 24h<br>[bpm]	78.30 ± 11.94<br>(70.00 – 88.00)	76.30 ± 12.06<br>(65.00 – 85.50)	0.530
ABPM SBPL/24h<br>[%]	39.54 ± 24.51<br>(20.00 – 57.00)	9.52 ± 7.71<br>(3.50 – 16.35)	<0.001
ABPM DBPL/24h<br>[%]	22.40 ± 18.39<br>(9.00 – 26.00)	6.24 ± 4.43<br>(1.00 – 9.50)	<0.001
ABPM SBP DIP<br>[%]	11.14 ± 5.19<br>(7.60 – 14.10)	10.30 ± 3.99<br>(7.80 – 12.40)	0.518
ABPM DBP DIP<br>[%]	16.58 ± 7.48<br>(11.00 – 21.10)	50.60 ± 7.21<br>(45.00 – 55.00)	0.645
AoSBP<br>[mm Hg]	111.03 ± 10.27<br>(102.00 – 117.33)	96.38 ± 7.80<br>(89.83 – 102.83)	<0.001
AoDBP<br>[mm Hg]	80.84 ± 10.53<br>(74.67 – 87.00)	67.75 ± 6.43<br>(63.00 – 71.33)	<0.001
AoMAP<br>[mm Hg]	95.48 ± 9.97<br>(87.67 – 101.33)	82.30 ± 6.58<br>(76.33 – 87.17)	<0.001
AoPP<br>[mm Hg]	30.19 ± 6.48<br>(25.67 – 35.33)	28.63 ± 4.35<br>(24.83 – 31.17)	0.328

SBP: systolic blood pressure; DBP: diastolic blood pressure; MAP: mean arterial pressure; PP: pulse pressure; ABPM: ambulatory blood pressure; HR: heart rate; bpm: beats per minute, SBPL: systolic blood pressure load; DBPL: diastolic blood pressure load; AoSBP: aortic (central) systolic blood pressure; AoDBP: aortic (central) diastolic blood pressure; AoMAP: aortic (central) mean blood pressure; AoPP: aortic (central) pulse pressure.
