# Supplementary material for: Serum Periostin as a Potential Biomarker in Pediatric Patients with Primary Hypertension
Source: J Clin Med. 2021 May 15;10(10):2138. doi: 10.3390/jcm10102138 (PMC8156565; doi:10.3390/jcm10102138)
Supplement: Supplementary file 1 [file jcm-10-02138-s001.zip › jcm-1192343-supplementary/Supplementary Table S3.pdf]

Journal of
Clinical Medicine

MDPI

Supplementary Table S3. Parameters of arterial structure and function in the study and the control group (data presented as mean ± standard deviation and interquartile range).

Parameter	Study group	Control group	*p*
aPWV<br>[m/s]	5.17 ± 0.93<br>(4.53 – 5.70)	4.49 ± 0.72<br>(3.90 – 4.83)	0.004
aPWV Z-score	0.02 ± 1.72<br>(−1.54 – 1.39)	−1.13 ± 1.36<br>(−2.34 – −0.11)	0.009
AIx75HR<br>[%]	−1.07 ± 13.66<br>(−11.00 – 5.00)	−3.77 ± 12.77<br>(−9.17 – 2.17)	0.938
Buckberg SEVR<br>[%]	161.40 ± 37.47<br>(133.33 – 189.00)	159.77 ± 30.19<br>(136.17 – 172.17)	0.815
cIMT<br>[mm]	0.45 ± 0.07<br>(0.40 – 0.50)	0.39 ± 0.03<br>(0.37 – 0.42)	<0.001
cIMT Z-score	1.30 ± 1.33<br>(0.29 – 2.44)	0.16 ± 0.57<br>(−0.20 – 0.63)	<0.001
ET beta	4.12 ± 3.53<br>(2.70 – 4.30)	3.71 ± 0.98<br>(3.15 – 4.55)	0.576
ET Ep<br>[kPa]	55.50 ± 45.74<br>(35.00 – 61.00)	44.20 ± 12.40<br>(34.50 – 53.00)	0.503
ET AC<br>[mm^2/kPa]	1.20 ± 0.53<br>(0.91 – 1.46)	1.06 ± 0.33<br>(0.83 – 1.22)	0.278
ET AIx<br>[%]	−2.08 ± 18.37<br>(−7.80 – 1.10)	−4.08 ± 7.57<br>(−7.95 – −1.30)	0.432
ET PWVbeta<br>[m/s]	4.22 ± 1.42<br>(3.40 – 4.70)	3.91 ± 0.55<br>(3.45 – 4.30)	0.435
ET D max<br>[mm]	6.31 ± 0.66<br>(5.94 – 6.70)	5.89 ± 0.74<br>(5.25 – 6.47)	0.023
ET D min<br>[mm]	5.44 ± 0.68<br>(5.04 – 5.89)	5.07 ± 0.68<br>(4.57 – 5.52)	0.041
ET DATmax<br>[ms]	133.82 ± 37.92<br>(107.00 – 146.00)	149.05 ± 48.78<br>(125.50 – 149.50)	0.219

aPWV: aortic pulse wave velocity; AIx75HR: augmentation index normalized to heart rate of 75 beats per minute; SEVR: subendocardial viability ratio; cIMT: common carotid artery intima-media thickness; ET: ECHO-tracking; beta: stiffness index; Ep: pressure strain elasticity modulus; AC: arterial compliance; AIx: augmentation index; D max: maximal diameter of the right common carotid artery; D min: minimal diameter of the right common carotid artery; DATmax: acceleration time to the right common carotid artery maximal diameter.
